# Supplementary material for: Collaborative and privacy-preserving retired battery sorting for profitable direct recycling via federated machine learning
Source: Nat Commun. 2023 Dec 5;14:8032. doi: 10.1038/s41467-023-43883-y (PMC10697957; doi:10.1038/s41467-023-43883-y)
Supplement: Supplementary file 1 — Supplementary Information [file 41467_2023_43883_MOESM1_ESM.pdf]

Supplementary information

Title

Collaborative and privacy-preserving retired battery sorting for profitable direct recycling via federated machine learning

Author list

Shengyu Tao[1#], Haizhou Liu[1#], Chongbo Sun[1#], Haocheng Ji[1], Guanjun Ji[1], Zhiyuan Han[1], Runhua Gao[1], Jun Ma[1], Ruifei Ma[1], Yuou Chen[1], Shiyi Fu[2], Yu Wang[2], Yaojie Sun[2], Yu Rong[3], Xuan Zhang[1*], Guangmin Zhou[1*] and Hongbin Sun[1,4,5*]

Affiliations

1. Tsinghua-Berkeley Shenzhen Institute, Tsinghua Shenzhen International Graduate School, Tsinghua University, China

2. School of Information Science and Technology, Fudan University, China

3. Tencent AI Lab, Tencent, China

4. Department of Electrical Engineering, Tsinghua University, China

5. College of Electrical and Power Engineering, Taiyuan University of Technology, China

[#] These authors contributed equally.

*Correspondence to whom should be addressed:

Email: xuanzhang@sz.tsinghua.edu.cn

Email: guangminzhou@sz.tsinghua.edu.cn

Email: shb@tsinghua.edu.cn

This file includes Supplementary Figures 1 to 13, Supplementary Tables 1 to 15 and Supplementary Notes 1 to 4.

Supplementary Figure 1 The typical discharging/discharging curve for each class of battery, after denoising and linear interpolation. Source data are provided as a Source Data file.

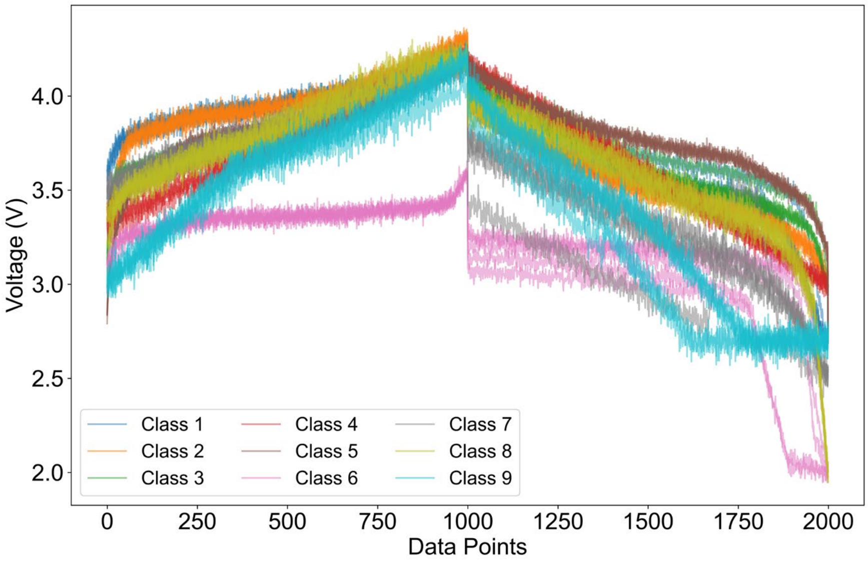

Supplementary Figure 2 The voltage-capacity curve in the charging cycle for the batteries. The unit for voltage is in volts, and the unit for capacity is in Ah. Different colors in each subplot represent unique batteries. The subplot title follows a format of A: B, which indicates the batteries originate from manufacturer A with cathode material B. Source data are provided as a Source Data file.

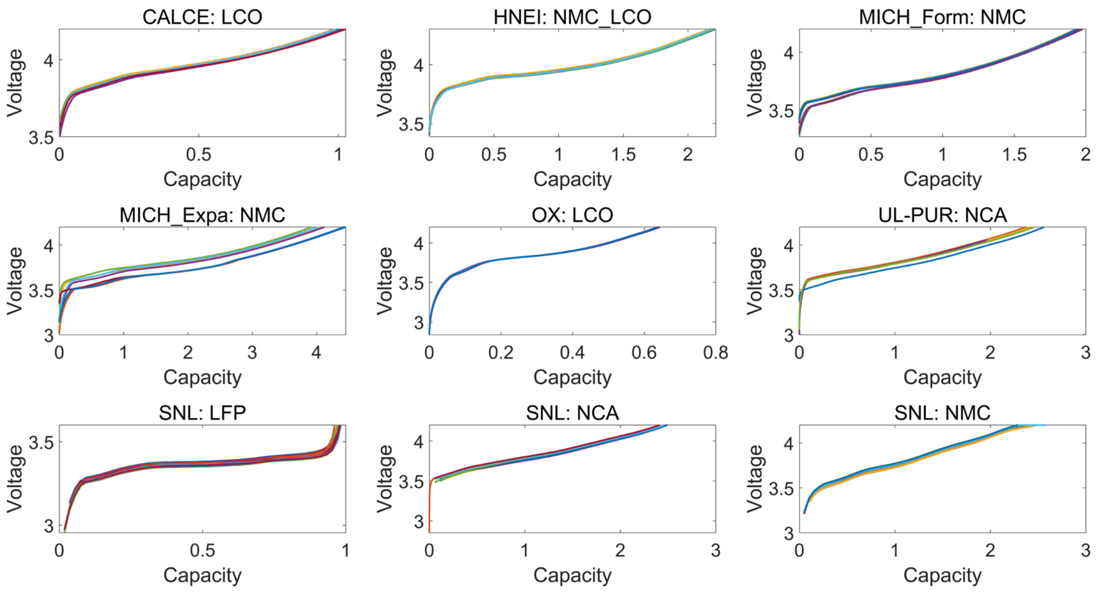

Supplementary Figure 3 The voltage-capacity curve in the discharging cycle for the batteries. The unit for voltage is in volts, and the unit for capacity is in Ah. Different colors in each subplot represent unique batteries. The subplot title follows a format of A: B, which indicates the batteries originate from manufacture A with cathode material B. Source data are provided as a Source Data file.

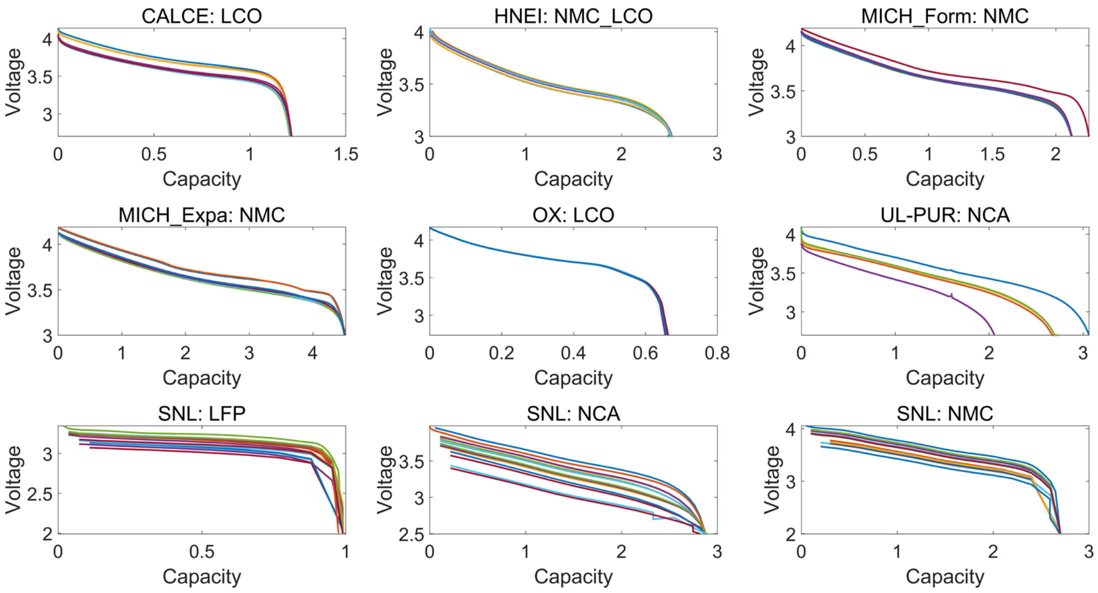

Supplementary Figure 4 The dQ/dV curve in the charging cycle for the batteries. The unit for voltage is in volts, and the unit for dQ/dV is in Ah/ volts. Different colors in each subplot represent unique batteries. The subplot title follows a format of A: B, which indicates the batteries originate from manufacture A with cathode material B. Source data are provided as a Source Data file.

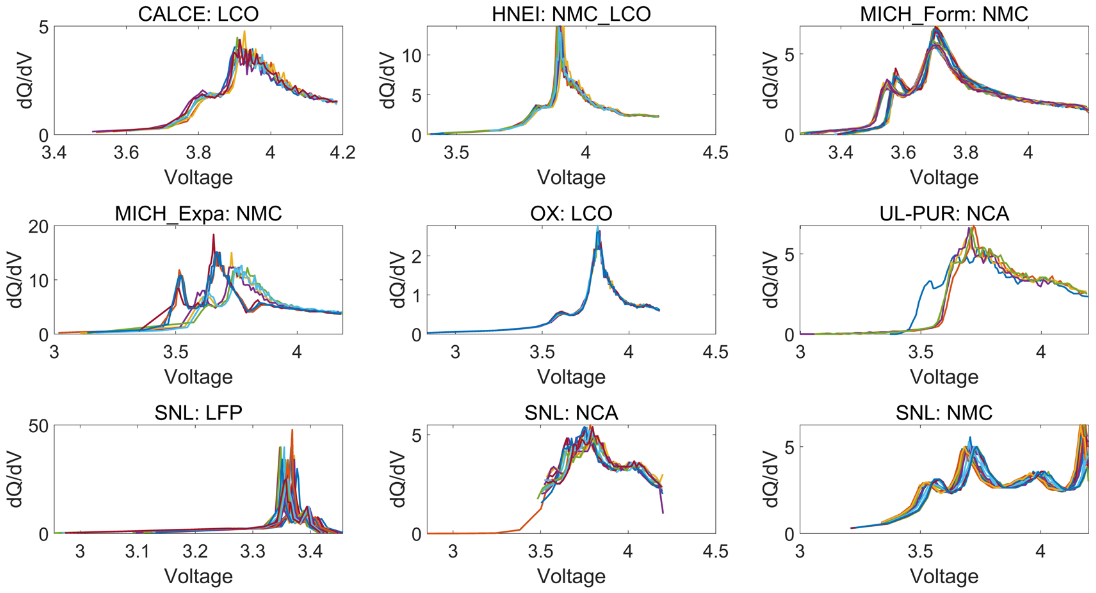

Supplementary Figure 5 The dQ/dV curve in the discharging cycle for the batteries. The unit for voltage is in volts, and the unit for dQ/dV is in Ah/ volts. Different colors in each subplot represent unique batteries. The subplot title follows a format of A: B, which indicates the batteries originate from manufacturer A with cathode material B. Source data are provided as a Source Data file.

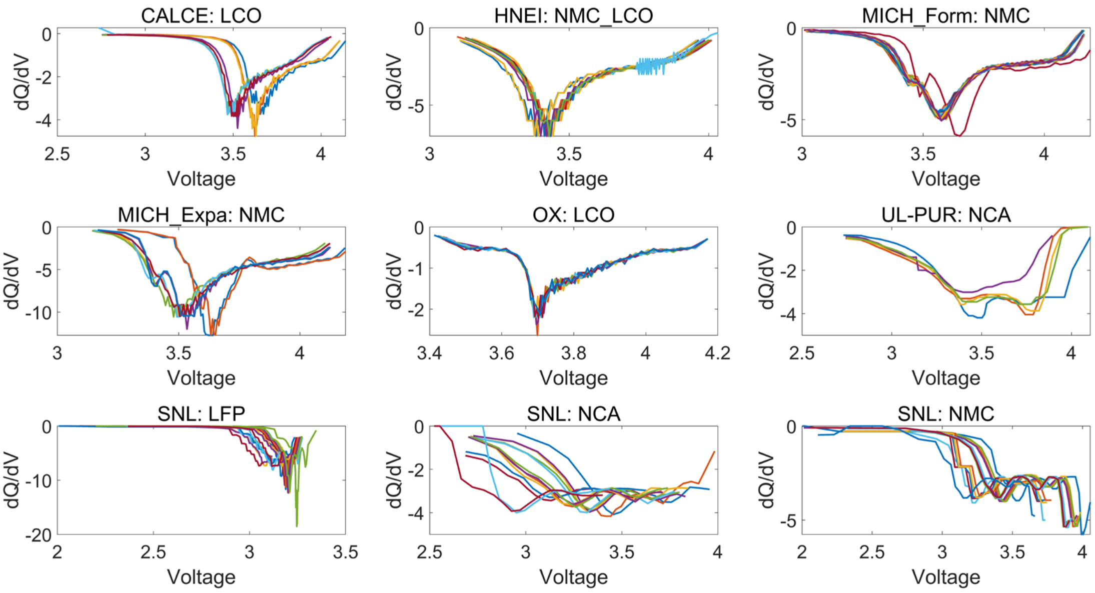

Supplementary Figure 6 The Pearson correlation matrix of the extracted features across nine classes of batteries. The red color (blue color) indicates the features are positively (negatively) correlated. The darker the color, the more correlation existed in the features. Source data are provided as a Source Data file.

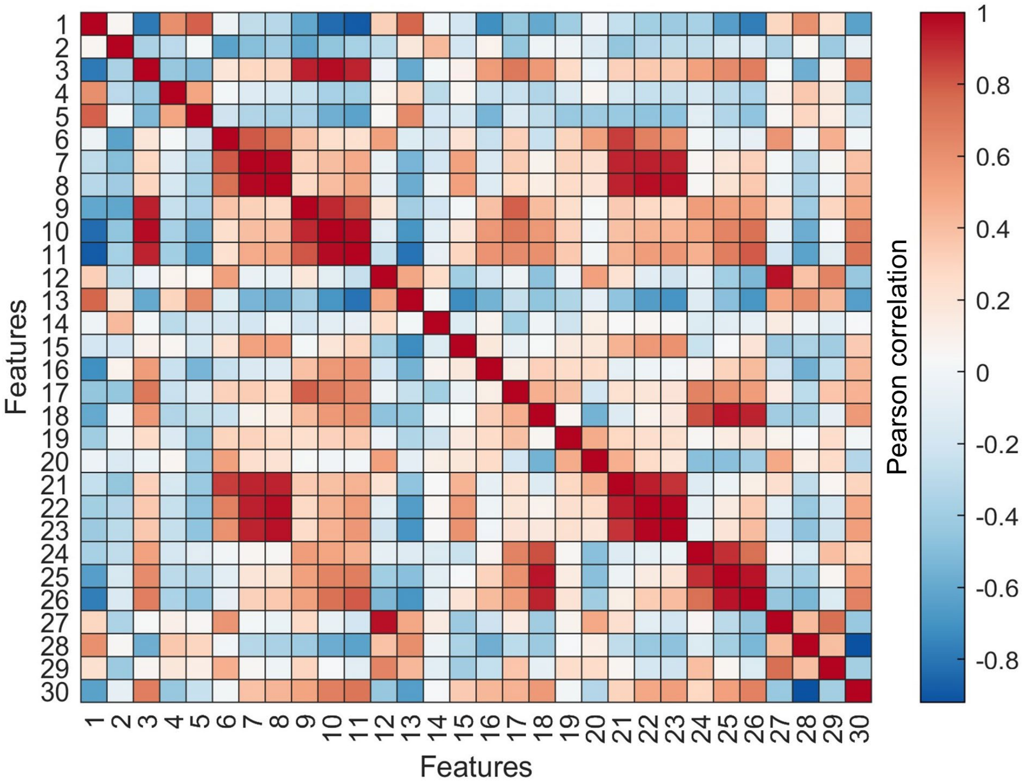

Supplementary Figure 7 The classification accuracy of the MV and WDV methods at selected random noise levels (NSR = 2%, 6%, and 10%), respectively. Source data are provided as a Source Data file.

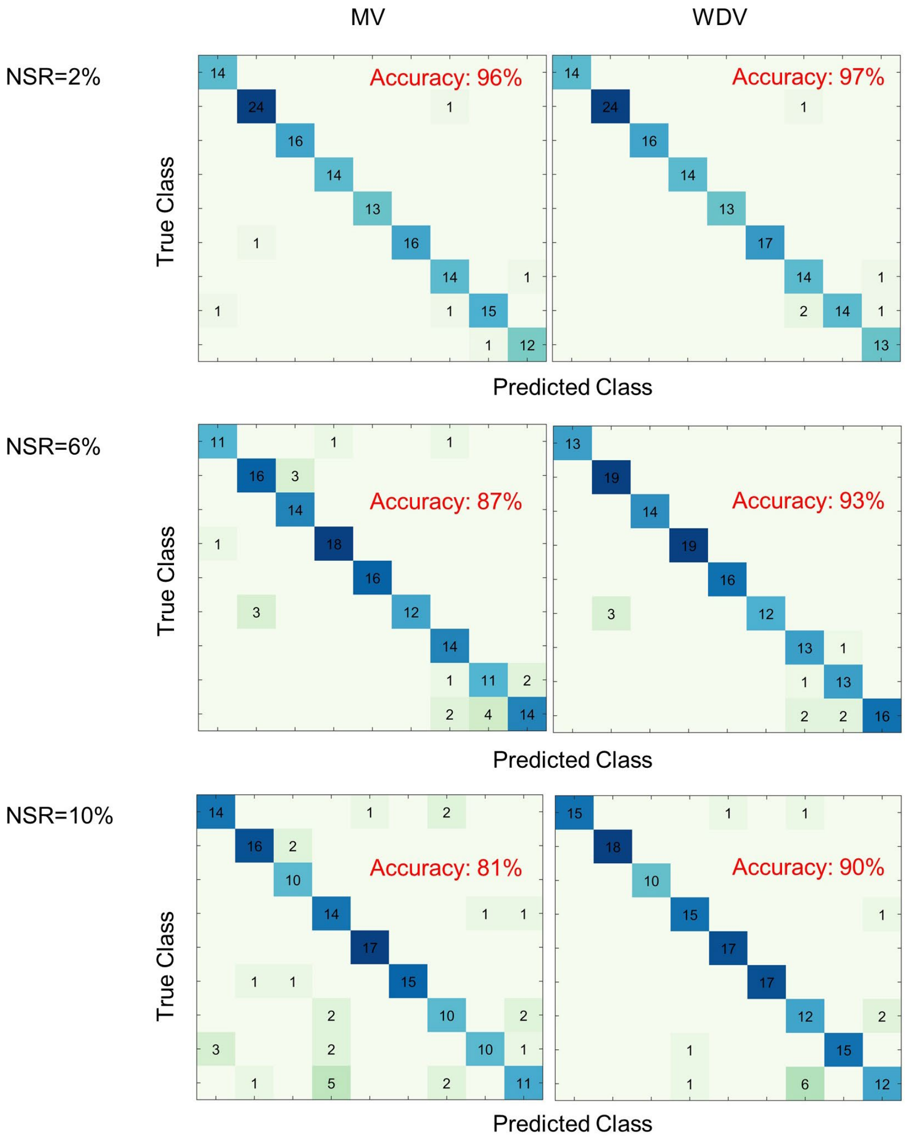

Supplementary Figure 8 The two-dimensional feature space spanned by the non-salient features. Each class is indicated as unique colors in the legend. Source data are provided as a Source Data file.

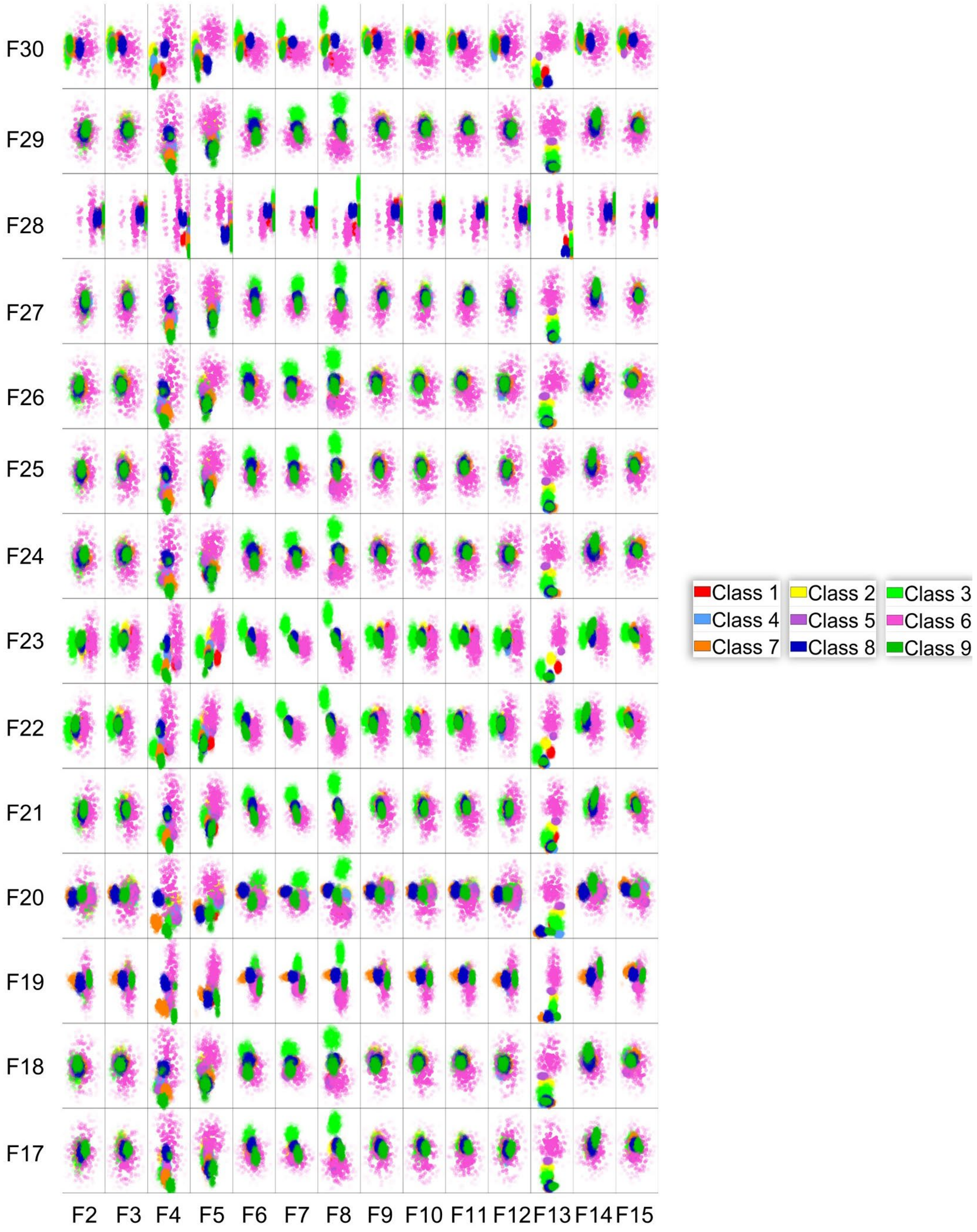

Supplementary Figure 9 The linear correlation between heterogeneity and classification accuracy under non-federated situations. The color bar indicates the accuracy value. Source data are provided as a Source Data file.

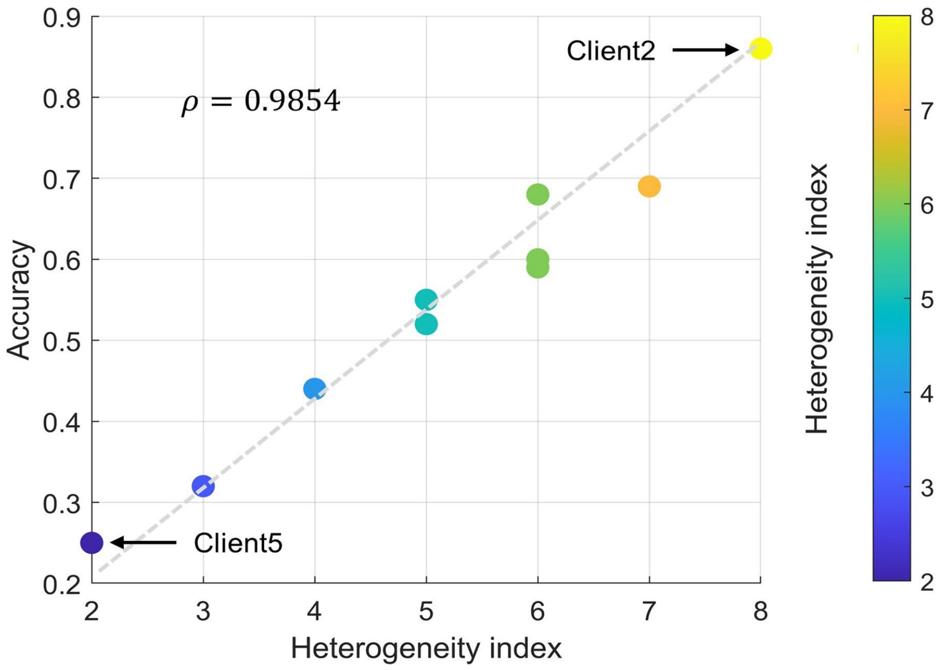

Supplementary Figure 10 The confusion matrix for the MV and WDV methods when the former achieves its best performance. Source data are provided as a Source Data file.

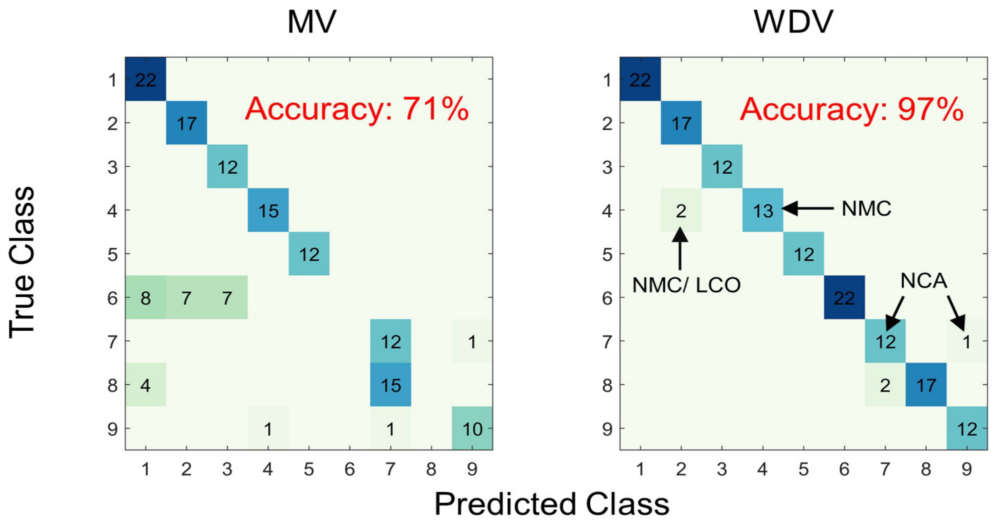

Supplementary Figure 11 The battery classification performance of a Convolutional Neural Networks (CNN) for comparative study. Up: confusion matrix of the classification results. Down: training time required for clients of different sample sizes (under Python 3.9.3). The CNN structure consists of two consecutive sets of Convolutional-MaxPooling-Dropout layers, followed by a fully-connected layer, a dropout layer, and a final fully-connected layer. Source data are provided as a Source Data file.

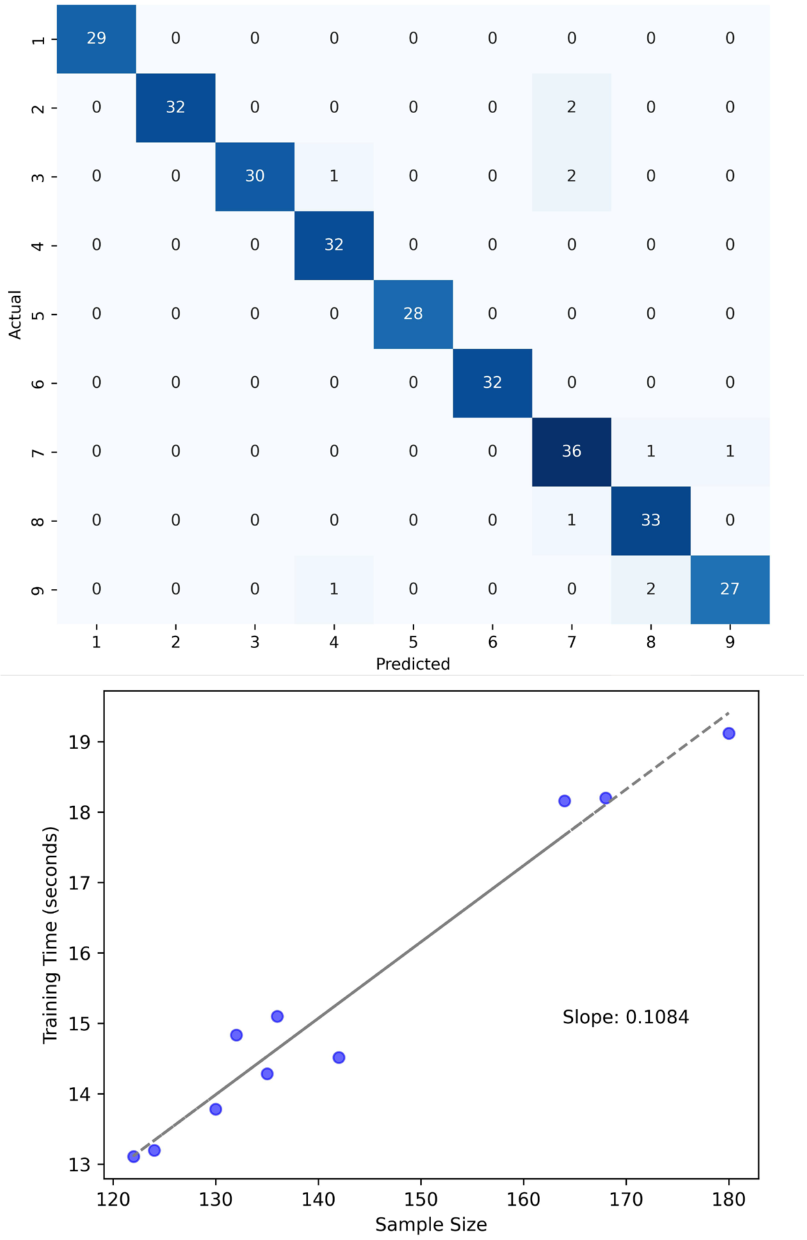

Supplementary Figure 12 The overall classification accuracy of the proposed WDV method under unrecoverable parameter loss and different heterogeneity settings, where the receiving end treats the lost client model sorting result as a random guess (a 0.5 sorting probability). The client data distribution is randomly generated with the same method described in the Client Simulation Section. Source data are provided as a Source Data file.

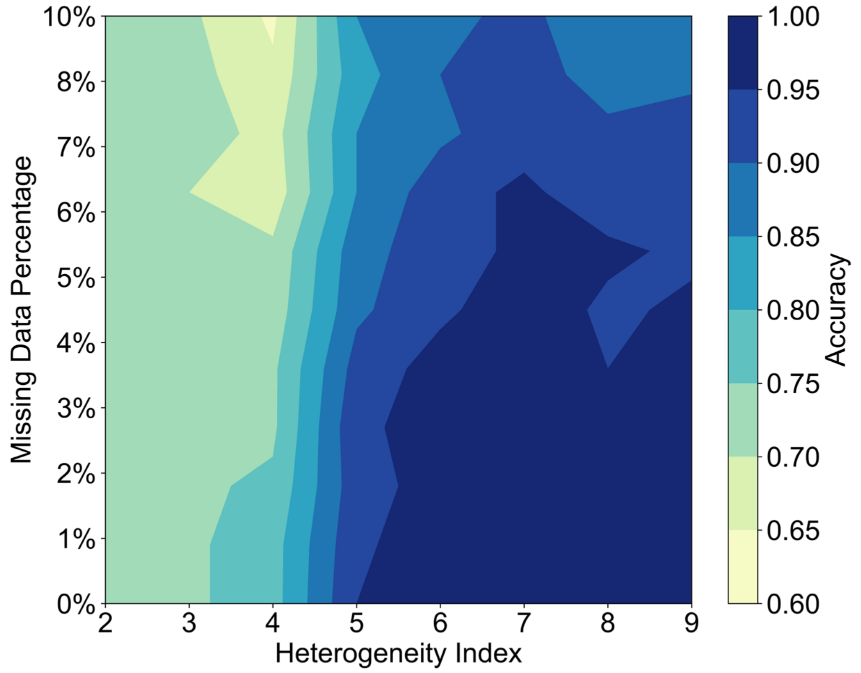

Supplementary Figure 13 Out-of-bag classification error of as-trained random forest models with different number of trees J. When J is set to 10, a balance is achieved between classification accuracy and computational cost, the latter of which is linearly dependent on the number of trees. Source data are provided as a Source Data file.

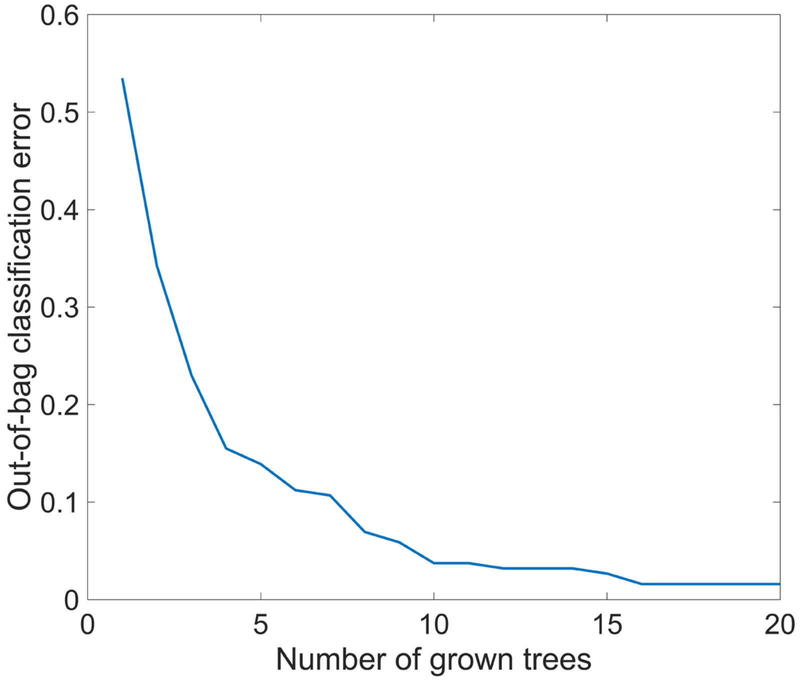

Supplementary Table 1 The classified battery groups and detailed information.

Manufacturers	Cathode	Class	Cells	Composition	Capacity
CALCE[1-5]	LCO	1	7	$\mathrm{LiCoO_2}$	1.35
HNEI[6]	NMC_LCO	2	13	$\mathrm{LiCoO_2}$ and $\mathrm{LiNi_4Co_4Mn_2O_2}$	2.80
MICH-Expa[7]	NMC	3	8	NMC111: CB: PVDF (94:3:3)	5.00
MICH-Form[8]	NMC	4	39	NMC111: C65: PVDF (94:3:3)	2.36
OX[9]	LCO	5	8	$\mathrm{LiCoO_2}$	0.74
SNL[10]	LFP	6	21	$\mathrm{LiFePO_4}$	1.10
SNL[10]	NCA	7	14	$\mathrm{LiNi}_x\mathrm{Co}_y\mathrm{Al}_{1-x-y}\mathrm{O}_2$	3.20
SNL[10]	NMC	8	15	$\mathrm{LiNi}_x\mathrm{Mn}_y\mathrm{Co}_{1-x-y}\mathrm{O}_2$	3.00
UL-PUR[11]	NCA	9	5	$\mathrm{LiNi_{0.8}Co_{0.15}Al_{0.05}O_2}$	3.40
Total			130		(Ah)

Supplementary Table 2 The explanation of the feature extraction.

Feature	Definition
F1(16)	The peak intensity of the dV/dQ curve
F2(17)	The time when the peak intensity of the dV/dQ curve arrives
F3(18)	The voltage where the peak intensity of the dV/dQ curve arrives
F4(19)	The kurtosis statistics of the dV/dQ curve
F5(20)	The skewness statistics of the dV/dQ curve
F6(21)	Q1 quantile of the voltage
F7(22)	Q2 quantile of the voltage
F8(23)	Q3 quantile of the voltage
F9(24)	Q1 quantile of the capacity
F10(25)	Q2 quantile of the capacity
F11(26)	Q3 quantile of the capacity
F12(27)	The kurtosis statistics of the voltage
F13(28)	The skewness statistics of the voltage
F14(29)	The kurtosis statistics of the capacity
F15(30)	The skewness statistics of the capacity

Note: The format of the extracted features follows Fa (Fb), which denotes that Fa and Fb are the features extracted from the charging and discharging process, respectively.

Supplementary Table 3 The detailed setting when clients have homogeneous data access.

Client	Class (Battery number)	Total
1	1 (10), 2 (15), 3 (7), 4 (18), 5 (17), 6 (10), 7 (10), 8 (12), 9 (10)	9 (109)
2	1 (18), 2 (17), 3 (15), 4 (19), 5 (16), 6 (16), 7 (15), 8 (19), 9 (11)	9 (146)
3	1 (11), 2 (12), 3 (11), 4 (20), 5 (14), 6 (12), 7 (9), 8 (9), 9 (8)	9 (106)
4	1 (16), 2 (18), 3 (14), 4 (17), 5 (15), 6 (12), 7 (14), 8 (12), 9 (10)	9 (128)
5	1 (13), 2 (14), 3 (21), 4 (14), 5 (20), 6 (19), 7 (25), 8 (23), 9 (21)	9 (170)
6	1 (27), 2 (16), 3 (16), 4 (19), 5 (16), 6 (18), 7 (19), 8 (19), 9 (19)	9 (169)
7	1 (15), 2 (20), 3 (19), 4 (21), 5 (18), 6 (13), 7 (23), 8 (17), 9 (12)	9 (158)
8	1 (21), 2 (19), 3 (25), 4 (20), 5 (17), 6 (17), 7 (18), 8 (17), 9 (12)	9 (166)
9	1 (24), 2 (12), 3 (25), 4 (15), 5 (19), 6 (25), 7 (15), 8 (26), 9 (18)	9 (179)
10	1 (20), 2 (11), 3 (12), 4 (17), 5 (11), 6 (6), 7 (15), 8 (11), 9 (11)	9 (114)
Total battery number		1445

Note: In the Class (Battery number) column, the value follows the format of A(b), which denotes each client has batteries from class A and the according numbers of batteries in this class are b, respectively. In the Total column, the value follows the format of C(d), which denotes that each client has a total number of C classes of batteries, and the total number of batteries in all class are d. Since we assume all clients have homogeneous data access, C=9 for all clients.

Supplementary Table 4 The privacy budgets (PBs) in each class when referenced at a 0.9 F1-score level.

Method	Non-federated	Federated	
Class	IL	MV	WDV
LCO (CALCE, Class1)	5	6	10
NMC\ LCO (HNEI, Class2)	5	5	10
NMC (MICH-Expa, Class3)	8	9	10
NMC (MICH-Form, Class4)	2	6	9
LCO (Ox, Class5)	10	10	10
LFP (SNL, Class6)	8	8	9
NCA (SNL, Class7)	2	2	5
NMC (SNL, Class8)	2	4	6
NCA (UL-PUR, Class9)	0	2	6
Average	4.67	5.78	8.33
Increased PB (%) using WDV	+ 78%	+ 44%	-

Supplementary Table 5 The detailed setting when clients have heterogeneous data access.

Client	Class (Battery number)	Total
1	2 (41), 3 (41), 4 (34), 5 (36), 6 (45)	5 (197)
2	1 (20), 2 (11), 4 (12), 5 (15), 6 (16), 7 (12), 8 (10), 9 (17)	8 (113)
3	1 (15), 3 (22), 4 (14), 5 (16), 7 (21), 9 (23)	6 (111)
4	2 (27), 3 (23), 5 (33), 6 (25), 7 (22), 9 (29)	6 (159)
5	2 (82), 8 (79)	2 (161)
6	2 (38), 5 (33), 6 (39), 8 (36), 9 (26)	5 (172)
7	2 (58), 4 (43), 8 (49), 9 (45)	4 (195)
8	1 (20), 2 (25), 3 (17), 5 (20), 6 (20), 7 (16)	6 (118)
9	1 (40), 3 (51), 9 (49)	3 (140)
10	1 (26), 2 (23), 3 (27), 4 (25), 5 (20), 7 (21), 9 (27)	7 (169)
Total battery number		1535

Note: In the Class (Battery number) column, the value follows the format of A(b), which denotes each client has batteries from class A and the according numbers of batteries in this class are b, respectively. In the Total column, the value follows the format of C(d), which denotes that each client has a total number of C classes of batteries, and the total number of batteries in all class are d.

Supplementary Table 6 Recovery rate of LFP and NMC batteries using different methods.

Method	Pyro (in %)		Hydro (in %)		ML-direct (in %)	
Type	LFP	NMC	LFP	NMC	LFP	NMC
Cathode materials	94	94	98	94	90	90
Anode materials	94	94	95	94	90	90
Cathode current collector	93	93	93	93	90	90
Anode current collector	93	93	93	93	90	90
Separator	96	93	93	93	95	95
Shell	98	98	98	98	98	98
Note: The data is collected from the literature[12].						

Supplementary Table 7 Partial cost of different recycling methods (¥).

Method	Pyro	Hydro	ML-direct
Battery disassembly	900	850	1000
Sewage treatment	800	990	700
Equipment depreciation	390	365	400
Transportation	500	500	500
Average labor	2000	2150	1564
Note: The data is collected from the literature[12].			

Supplementary Table 8 List of material prices.

Material	Price (¥)	Source
Li_2CO_3	273000	https://dianchizhijia.com/
$NiSO_4$	32700	https://dianchizhijia.com/
$CoSO_4$	36300	https://dianchizhijia.com/
$MnSO_4$	6100	https://dianchizhijia.com/
Graphite	25800	https://dianchizhijia.com/
Al	18400	https://dianchizhijia.com/
Cu	65500	https://dianchizhijia.com/
$LiFePO_4$	81500	https://dianchizhijia.com/
$FePO_4$	13000	https://dianchizhijia.com/
$Li(Ni_{0.5}C_{0.2}M_{0.3})O_2$	230000	https://dianchizhijia.com/
Coke	2074	http://quote.eastmoney.com/center/futures.html
H_2SO_4	550	www.100ppi.com
NaOH	2000	www.100ppi.com
N_2	800	www.100ppi.com
Liquid ammonia	3800	www.100ppi.com
Degraded LFP battery	20000	https://dianchizhijia.com/
Degraded LFP cathode powder	38750	https://dianchizhijia.com/
Degraded NMC battery	39000	https://dianchizhijia.com/
Degraded NMC cathode powder	115000	https://dianchizhijia.com/
Note: The update time is May 22, 2023.		

Supplementary Table 9 Composition ratio of LFP and NMC batteries.

Battery Type	NMC	LFP
Cathode	0.26	0.25
Anode	0.15	0.13
Cathode current collector	0.07	0.06
Anode current collector	0.17	0.1
Electrolyte	0.1	0.16
Separator	0.22	0.27
Shell	0.03	0.03
Note: The data is collected from the literature[13].		

Supplementary Table 10 Cost analysis of LFP and NMC batteries using different methods (individual).

Method	Pyro (¥)		Hydro (¥)		ML-direct (¥)	
Type	LFP	NMC	LFP	NMC	LFP	NMC
Raw material	9687.50	29900.00	9687.50	29900.00	9687.50	29900.00
Reagent	637.49	951.35	326.26	468.35	3196.19	5410.20
Average labor	3400.00	3400.00	3500.00	3500.00	3064.00	3064.00
Electricity & Water	1360.00	1360.00	1960.00	1960.00	856.00	856.00
Equipment depreciation	390.00	390.00	365.00	365.00	400.00	400.00
Sewage treatment	800.00	800.00	990.00	990.00	700.00	700.00
Total	16274.99	36801.35	16828.76	37183.35	17903.69	40330.20

Note:
Individual means there is only one type of battery in one recycling process, and the battery cathode type was determined by historical information assumed. The data were collected from the literatures[12,14].

Supplementary Table 11 Revenue analysis of LFP and NMC batteries using different methods (individual).

Method	Pyro (¥)		Hydro (¥)		ML-direct (¥)	
Type	LFP	NMC	LFP	NMC	LFP	NMC
Li_2CO_3	12017.68	20425.60	12529.07	20425.60	\	\
$NiSO_4$	\	6403.32	\	6403.32	\	\
$CoSO_4$	\	2847.72	\	2847.72	\	\
$MnSO_4$	\	699.33	\	699.33	\	\
Li $(Ni_{0.5}C_{0.2}M_{0.3})O_2$	\	\	\	\	\	53831.15
$FePO_4$	\	\	3044.89	\	\	\
$LiFePO_4$	\	\	\	\	18337.50	\
Graphite	\	\	4421.30	5047.80	4188.60	5262.60
Al	1026.72	1197.84	1026.72	1197.84	993.60	1159.20
Cu	6091.50	10355.55	6091.50	10355.55	7663.50	10021.50
Total	19135.90	41929.37	27113.48	46977.17	31183.20	70274.45

Note:
Individual means there is only one type of battery in one recycling process, and the battery cathode type was determined by historical information assumed.

Supplementary Table 12 Cost, revenue, and profit of retired battery recycling (individual).

Methods	Type	Cost (¥)	Revenue (¥)	Profit (¥)
Pyro	LFP	16274.99	19135.90	2860.91
	NMC	36801.35	41929.37	5128.02
Hydro	LFP	16828.76	27113.48	10284.72
	NMC	37183.35	46977.17	9793.82
ML-direct	LFP	17903.69	31183.20	13279.51
	NMC	40330.20	70274.45	29944.25
Note:<br>Individual means there is only one type of battery in one recycling process, and the battery cathode type was determined by historical information assumed.<br>profit = revenue - cost				

Supplementary Table 13 The influence of predict accuracy towards the production of ML-direct recycling (hybrid).

Ideal production	Accuracy (%)	Actual production	Equivalent price (¥)
$Li\ (Ni_{0.5}C_{0.2}M_{0.3})\ O_2$	Fed-WDV (97)	$Li\ (Ni_{0.5}C_{0.2}M_{0.3})\ O_2$	230000
	Fed-MV (71)	71 % $Li(Ni_{0.5}C_{0.2}M_{0.3})O_2$<br>+<br>29 % $LiFePO_4$	92887.5
	IL (55)	55 % $Li(Ni_{0.5}C_{0.2}M_{0.3})O_2$<br>+<br>45 % $LiFePO_4$	80687.5
$LiFePO_4$	Fed-WDV (97)	$LiFePO_4$	81500
	Fed-MV (71)	70 % $LiFePO_4$<br>+<br>30 % $Li(Ni_{0.5}C_{0.2}M_{0.3})O_2$	60862.5
	IL (55)	55 % $LiFePO_4$<br>+<br>45 % $Li(Ni_{0.5}C_{0.2}M_{0.3})O_2$	73062.5

Note:

Hybrid means there are multiple types of battery in one recycling process, and the battery cathode types were determined by our machine learning method, leveraging field information.

Fed-WDV, Fed-MV, and IL stand for federated machine learning using the MDV method (our work), federated machine learning using the MV method, and non-federated independent learning.

The impure product is regarded as degraded cathode material, which needs to be treated by hydrometallurgy again. We assumed the equivalent price is calculated by the sum of the product of accuracy and retired cathode powder price. For example, for the accuracy of 0.71 for NMC, the equivalent price = 0.71*115000+0.29*38750=92887.5.

Supplementary Table 14 The influence of prediction accuracy on the revenue of ML-direct recycling (hybrid).

Accuracy(%)	MR	Cathode (¥)	Others (¥)	Revenue (¥)
Fed-WDV(97)	0.00	53831.15	16443.30	70274.45
Fed-WDV(97)	0.33			57244.03
Fed-WDV(97)	0.50			50728.83
Fed-WDV(97)	0.67			44213.62
Fed-WDV(97)	1	18337.50	12845.70	31183.20
Fed-MV(71)	0.00	21634.54	15400.00	37034.53
Fed-MV(71)	0.33			33984.09
Fed-MV(71)	0.50			32458.88
Fed-MV(71)	0.67			30933.66
Fed-MV(71)	1	13994.21	13889.00	27883.22
IL(55)	0.00	18723.94	14824.38	33548.32
IL(55)	0.33			32822.02
IL(55)	0.50			32458.88
IL(55)	0.67			32095.73
IL(55)	1	16904.81	14464.62	31369.43

Note:

Hybrid means there are multiple types of battery in one recycling process, and the battery cathode types were determined by our machine learning method, leveraging field information.

Mixed ratio, MR = LFP/(LFP+NMC)

Fed-WDV, Fed-MV, and IL stand for federated machine learning using the MDV method (our work), federated machine learning using the MV method, and non-federated independent learning.

We assumed the revenue is calculated by the sum of the product of the cathode (weight, accuracy, and retired cathode powder price) and others (anode and current collector).

For example, for the accuracy of 0.71 for the LFP/(LFP+NMC) ratio of 0.5,

the revenue =

0.90*0.5*(0.25*38750.00+0.26*115000.00)+0.50*(4188.60+993.60+7663.50)+0.50*(5262.60+1159.20+10021.50)= 32458.88.

Supplementary Table 15 Cost, revenue, and profit of LFP and NMC batteries using different methods (hybrid).

Method	MR	Accuracy (%)	Cost (¥)	Revenue (¥)	Profit (¥)
Pyro	0.33	\	29959.23	34331.54	4372.32
	0.5	\	26538.17	30532.63	3994.46
	0.66	\	23117.11	26733.72	3616.61
Hydro	0.33	\	30398.48	40355.94	9957.45
	0.5	\	27006.05	37045.32	10039.27
	0.66	\	23613.62	33734.71	10121.09
ML-direct	0.33	Fed-WDV(97)	32854.70	57244.03	24389.33
		Fed-MV(71)	30686.80	33984.09	3297.29
		IL(55)	29490.72	32822.02	3331.30
	0.5	Fed-WDV(97)	29116.95	50728.83	21611.88
		Fed-MV(71)	29116.95	32458.88	3341.93
		IL(55)	29116.95	32458.88	3341.93
	0.66	Fed-WDV(97)	25379.19	44213.62	18834.42
		Fed-MV(71)	27547.09	30933.66	3386.56
		IL(55)	28743.17	32095.73	3352.56

Note:
Hybrid means there are multiple types of battery in one recycling process, and the battery cathode types were determined by our machine learning method, leveraging field information.
Mixed ratio, MR = LFP/(LFP+NMC)
Fed-WDV, Fed-MV, and IL stand for federated machine learning using the MDV method (our work), federated machine learning using the MV method, and non-federated independent learning.
We assumed the cost is calculated by the sum of the product of individual cost and battery ratio. For example, for the LFP/(LFP+NMC) ratio of 0.5 using ML-direct recycling, the cost = 0.5*17903.69+0.5*40330.20=29916.95.

Supplementary Note 1 The definition of the standardization of the retired batteries

Recyclers often face difficulty identifying the type of retired battery, given poor access to the full historical operating data. However, battery recyclers need information on the type of retired batteries to decide on the design of recycling strategies. Therefore, it is necessary to standardize the retired battery data, even though the battery type and historical usages are diversified. Our proposed standardized process aims to test retired batteries with a commonly accessible method and to obtain information about retired batteries. Specifically, battery information is represented by a voltage-capacity curve and a dQ/dV curve derived from the charging and discharging data, respectively. To obtain such curves, battery recyclers need to encourage the collaborators to charge, and discharge collected retired batteries for one cycle. The observed data on the charging and discharging characteristics, after being recorded, are preprocessed according to the unanimous protocol distributed by the recyclers to generate curves. The protocol first denoises measurement data by (a) identifying out-of-cycle missing data entries and filling them with their nearest non-missing data entries; (b) identifying in-cycle outlier data entries (defined as entries outside 3 times the median-absolute deviation) and replacing them with their nearest non-outlier data entries; and (c) performing median filtering based on 20 neighboring data (the neighboring size for SNL and HNEI dataset is 30 and 25, respectively) entries to smooth the data. Next, the protocol interpolates and differentiates the denoised data to yield the voltage capacity and dQ/dV curves, with a recommended interpolation length of 1000 data points. Based on the data standardization results, feature engineering can be conducted by extracting 30 key statistical features from the generated characteristic curves. It is assumed that batteries are mandatorily decommissioned when they reach a given threshold, i.e., 80% of the state of health (defined as the ratio of the current capacity to the nominal capacity), and are recovered by recyclers. Therefore, data standardization here is equivalent to obtaining information on the operating status of retired batteries at the end of their life with no requirements on the historical operational data. It is noted that the decommission threshold in this work is set as 90% of the nominal capacity of each battery due to the limited sample size of the batteries that are deemed to be decommissioned.

Also, as suggested in the main text, we deliberately retain *human-induced* and *cathode-heterogeneity-induced noises* with the intention of making the model robust and insensitive to these two types of noise. *The human-induced noise* arises from variations in parameter settings in feature engineering, as clients may decide on these settings based on the type of battery material. *Heterogeneity-induced noises* stem from the possibility that a client may have batteries of multiple cathode material types, complicating the data distribution. Consequently, our federated machine learning framework can be broadly applicable to various cathode material sorting under both homogenous and heterogenous data scenarios even if allowing the collaborators to preprocess the raw data flexibly, showcasing the potential for scalable industry implementation.

Supplementary Note 2

The definition of the quantiles:

The Q1 quantile splits off the lowest 25% of data from the highest 75%.

The Q2 quantile cuts the data set in half.

The Q3 quantile splits off the highest 25% of data from the lowest 75%.

Kurtosis is a measure of how outlier-prone a distribution is. The kurtosis of the normal distribution equals 3. When a distribution is more outlier-prone than the normal distribution, the kurtosis is larger than 3. When a distribution is less outlier-prone than the normal distribution, the kurtosis is smaller than 3. The definition of kurtosis:

$$k = \frac{\frac{1}{n}\sum_{i=1}^{n}(x_i - \bar{x})^4}{\left(\frac{1}{n}\sum_{i=1}^{n}(x_i - \bar{x})^2\right)^2} \tag{S1}$$

where x_i is the i-th feature point, $\bar{x}$ is the mean value of the feature vector, and n is the number of points in the feature vector.

Skewness is a measure of the asymmetry of the data around the sample mean. If skewness is negative, the data spread out more to the left of the mean than to the right. If skewness is positive, the data spread out more to the right. The definition of skewness:

$$s = \frac{\frac{1}{n}\sum_{i=1}^{n}(x_i - \bar{x})^3}{\left(\sqrt{\frac{1}{n}\sum_{i=1}^{n}(x_i - \bar{x})^2}\right)^3} \tag{S2}$$

where x_i is the i-th feature point, $\bar{x}$ is the mean value of the feature vector, and n is the number of points in the feature vector.

Supplementary Note 3

In contrast to the homogeneous data distribution among clients, the heterogeneous data distribution is more compatible with the real-world situation. We consider this situation by running the Client simulation process when setting the NC_k to 2. It means the minimum number of battery classes, namely the heterogeneity index in each client, is two to nine. The possible combinations of the clients under different random situations are also considered using the Monte Carlo method. Specifically, the Client simulation process is run with 50 Monte Carlo trials. The random seed is retrieved using a sequence from 50 to 100 in MATLAB 2022a with the “v5uniform” method. Since we simulate the heterogeneity index from two to nine, there are in total of 400 trials in our heterogeneous data distribution setting. For each trial, the number of classes and batteries in one specific class are randomly shuffled. The model performance evaluations are conducted for each trial.

Supplementary Note 4

This part aims to describe the calculative process of economical evaluation.

Step 1:

Supplementary Tables 6, 7, 8, and 9 are basic data lists.

Step 2:

Supplementary Tables 10, 11 are calculation results based on the basic data[12,14] in Step 1.

Step 3:

Supplementary Table 12 is a statistical result of Supplementary Tables 10, 11 in Step 2.

Step 4:

Supplementary Tables 13, 14, and 15 demonstrate the calculation process.

Supplementary References

1 Zheng, F. *et al.* Influence of different open circuit voltage tests on state of charge online estimation for lithium-ion batteries. *Appl. Energ.* **183**, 513-525 (2016). https://doi.org:https://doi.org/10.1016/j.apenergy.2016.09.010

2 Xing, Y., He, W., Pecht, M. & Tsui, K. L. State of charge estimation of lithium-ion batteries using the open-circuit voltage at various ambient temperatures. *Appl. Energ.* **113**, 106-115 (2014). https://doi.org:https://doi.org/10.1016/j.apenergy.2013.07.008

3 He, W., Williard, N., Osterman, M. & Pecht, M. Prognostics of lithium-ion batteries based on Dempster–Shafer theory and the Bayesian Monte Carlo method. *J. Power Sources* **196**, 10314-10321 (2011). https://doi.org:https://doi.org/10.1016/j.jpowsour.2011.08.040

4 Xing, Y., Ma, E. W. M., Tsui, K.-L. & Pecht, M. An ensemble model for predicting the remaining useful performance of lithium-ion batteries. *Microelectron. Reliab.* **53**, 811-820 (2013). https://doi.org:https://doi.org/10.1016/j.microrel.2012.12.003

5 Saxena, S., Hendricks, C. & Pecht, M. Cycle life testing and modeling of graphite/LiCoO2 cells under different state of charge ranges. *J. Power Sources* **327**, 394-400 (2016). https://doi.org:https://doi.org/10.1016/j.jpowsour.2016.07.057

6 Devie, A., Baure, G. & Dubarry, M. Intrinsic Variability in the Degradation of a Batch of Commercial 18650 Lithium-Ion Cells. *Energies* **11**, 1031 (2018).

7 Mohtat, P., Lee, S., Siegel, J. B. & Stefanopoulou, A. G. Reversible and Irreversible Expansion of Lithium-Ion Batteries Under a Wide Range of Stress Factors. *J. Electrochem. Soc.* **168**, 100520 (2021). https://doi.org:10.1149/1945-7111/ac2d3e

8 Weng, A. *et al.* Predicting the impact of formation protocols on battery lifetime immediately after manufacturing. *Joule* **5**, 2971-2992 (2021). https://doi.org:https://doi.org/10.1016/j.joule.2021.09.015

9 Birkl, C. (University of Oxford, 2017). https://ora.ox.ac.uk/objects/uuid:03ba4b01-cfed-46d3-9b1a-7d4a7bdf6fac

10 Preger, Y. *et al.* Degradation of Commercial Lithium-Ion Cells as a Function of Chemistry and Cycling Conditions. *J. Electrochem. Soc.* **167**, 120532 (2020). https://doi.org:10.1149/1945-7111/abae37

11 Juarez-Robles, D., Jeevarajan, J. A. & Mukherjee, P. P. Degradation-Safety Analytics in Lithium-Ion Cells: Part I. Aging under Charge/Discharge Cycling. *J. Electrochem. Soc.* **167**, 160510 (2020). https://doi.org:10.1149/1945-7111/abc8c0

12 Ji, G. *et al.* Direct regeneration of degraded lithium-ion battery cathodes with a multifunctional organic lithium salt. *Nat. Commun.* **14**, 584 (2023). https://doi.org:10.1038/s41467-023-36197-6

13 Jung, J. C.-Y., Sui, P.-C. & Zhang, J. A review of recycling spent lithium-ion battery cathode materials using hydrometallurgical treatments. *J. Energy Storage* **35**, 102217 (2021). https://doi.org:https://doi.org/10.1016/j.est.2020.102217

14 Qian, G. *et al.* Value-creating upcycling of retired electric vehicle battery cathodes. *Cell Rep. Phys. Sci.* **3**, 100741 (2022). https://doi.org:https://doi.org/10.1016/j.xcrp.2022.100741
