# Supplementary material for: Collaborative and privacy-preserving retired battery sorting for profitable direct recycling via federated machine learning
Source: Nat Commun. 2023 Dec 5;14:8032. doi: 10.1038/s41467-023-43883-y (PMC10697957; doi:10.1038/s41467-023-43883-y)
Supplement: Supplementary file 3 — Reporting Summary [file 41467_2023_43883_MOESM3_ESM.pdf]

nature portfolio

Corresponding author(s): NCOMMS-23-27860B

Last updated by author(s): Oct 25, 2023

# Reporting Summary

Nature Portfolio wishes to improve the reproducibility of the work that we publish. This form provides structure for consistency and transparency in reporting. For further information on Nature Portfolio policies, see our Editorial Policies and the Editorial Policy Checklist.

## Statistics

For all statistical analyses, confirm that the following items are present in the figure legend, table legend, main text, or Methods section.

| n/a | Confirmed | |
|---|---|---|
| ☐ | ☒ | The exact sample size (*n*) for each experimental group/condition, given as a discrete number and unit of measurement |
| ☐ | ☒ | A statement on whether measurements were taken from distinct samples or whether the same sample was measured repeatedly |
| ☒ | ☐ | The statistical test(s) used AND whether they are one- or two-sided<br>*Only common tests should be described solely by name; describe more complex techniques in the Methods section.* |
| ☒ | ☐ | A description of all covariates tested |
| ☒ | ☐ | A description of any assumptions or corrections, such as tests of normality and adjustment for multiple comparisons |
| ☒ | ☐ | A full description of the statistical parameters including central tendency (e.g. means) or other basic estimates (e.g. regression coefficient) AND variation (e.g. standard deviation) or associated estimates of uncertainty (e.g. confidence intervals) |
| ☒ | ☐ | For null hypothesis testing, the test statistic (e.g. *F*, *t*, *r*) with confidence intervals, effect sizes, degrees of freedom and *P* value noted<br>*Give P values as exact values whenever suitable.* |
| ☒ | ☐ | For Bayesian analysis, information on the choice of priors and Markov chain Monte Carlo settings |
| ☒ | ☐ | For hierarchical and complex designs, identification of the appropriate level for tests and full reporting of outcomes |
| ☒ | ☐ | Estimates of effect sizes (e.g. Cohen's *d*, Pearson's *r*), indicating how they were calculated |

*Our web collection on statistics for biologists contains articles on many of the points above.*

## Software and code

Policy information about availability of computer code

| | |
|---|---|
| Data collection | The data used in the study is all from open-source dataset described in the data availability section. |
| Data analysis | MATLAB 2022a is used to perform the modeling work. |

For manuscripts utilizing custom algorithms or software that are central to the research but not yet described in published literature, software must be made available to editors and reviewers. We strongly encourage code deposition in a community repository (e.g. GitHub). See the Nature Portfolio guidelines for submitting code & software for further information.

## Data

Policy information about availability of data

All manuscripts must include a data availability statement. This statement should provide the following information, where applicable:

- Accession codes, unique identifiers, or web links for publicly available datasets
- A description of any restrictions on data availability
- For clinical datasets or third party data, please ensure that the statement adheres to our policy

The Center for Advanced Life Cycle Engineering (CALCE), Hawaii Natural Energy Institute (HNEI), University of Michigan (MICH), University of Oxford (OX), the Sandia National Laboratories (SNL) and Underwriters Laboratories–Purdue University (UL-PUR) datasets used in this study are available at www.batteryarchive.org . For the full details of the dataset and policies for data reuse, please refer to their website, respectively.

## Research involving human participants, their data, or biological material

Policy information about studies with human participants or human data. See also policy information about sex, gender (identity/presentation), and sexual orientation and race, ethnicity and racism.

| | |
|---|---|
| Reporting on sex and gender | no sex and gender issue considered in this work. |
| Reporting on race, ethnicity, or other socially relevant groupings | no race, ethnicity, or other socially relevant issue considered in this work. |
| Population characteristics | This information is not considered in this work. |
| Recruitment | This information is not considered in this work. |
| Ethics oversight | This information is not considered in this work. |

Note that full information on the approval of the study protocol must also be provided in the manuscript.

# Field-specific reporting

Please select the one below that is the best fit for your research. If you are not sure, read the appropriate sections before making your selection.

☐ Life sciences ☐ Behavioural & social sciences ☒ Ecological, evolutionary & environmental sciences

For a reference copy of the document with all sections, see nature.com/documents/nr-reporting-summary-flat.pdf

# Ecological, evolutionary & environmental sciences study design

All studies must disclose on these points even when the disclosure is negative.

| | |
|---|---|
| Study description | we use federated machine learning to sort the retired batteries. factorized research structure. 200 Monte Carlo experiments were carried out. |
| Research sample | the research data are all from the open source data set. |
| Sampling strategy | the data in the last cycle are used due to the problem setting that we do not have any access to historical use information. |
| Data collection | the research data are all from the open source data set. The Center for Advanced Life Cycle Engineering (CALCE), Hawaii Natural Energy Institute (HNEI), University of Michigan (MICH), University of Oxford (OX), the Sandia National Laboratories (SNL) and Underwriters Laboratories–Purdue University (UL-PUR) datasets used in this study are available at www.batteryarchive.org . |
| Timing and spatial scale | For the full details of the dataset and policies for data reuse, please refer to their website for The Center for Advanced Life Cycle Engineering (CALCE), Hawaii Natural Energy Institute (HNEI), University of Michigan (MICH), University of Oxford (OX), the Sandia National Laboratories (SNL) and Underwriters Laboratories–Purdue University (UL-PUR) datasets, respectively. |
| Data exclusions | the data in the last cycle are used due to the problem setting that we do not have any access to historical use information. other data are not used. |
| Reproducibility | by setting the random seed in MATLAB2022a to ensure reproducibility. |
| Randomization | not random, the data is provided from different dataset thus no randomization. |
| Blinding | not involved in this study. |

Did the study involve field work? ☐ Yes ☒ No

# Reporting for specific materials, systems and methods

We require information from authors about some types of materials, experimental systems and methods used in many studies. Here, indicate whether each material, system or method listed is relevant to your study. If you are not sure if a list item applies to your research, read the appropriate section before selecting a response.

## Materials & experimental systems

| n/a | Involved in the study |
| --- | --- |
| ☒ | ☐ Antibodies |
| ☒ | ☐ Eukaryotic cell lines |
| ☒ | ☐ Palaeontology and archaeology |
| ☒ | ☐ Animals and other organisms |
| ☒ | ☐ Clinical data |
| ☒ | ☐ Dual use research of concern |
| ☒ | ☐ Plants |

## Methods

| n/a | Involved in the study |
| --- | --- |
| ☒ | ☐ ChIP-seq |
| ☒ | ☐ Flow cytometry |
| ☒ | ☐ MRI-based neuroimaging |
